# A flow cytometry‐based assay to determine the phagocytic activity of both clinical and nonclinical antibody samples against *Chlamydia trachomatis*

**DOI:** 10.1002/cyto.a.23353

**Published:** 2018-03-07

**Authors:** Marco Grasse, Ida Rosenkrands, Anja Olsen, Frank Follmann, Jes Dietrich

**Affiliations:** ^1^ Department of Infectious Disease Immunology Statens Serum Institut Copenhagen Denmark; ^2^ Department of Immunology, Institute for Biomedical Aging Research Universität Innsbruck Innsbruck Austria

**Keywords:** chlamydia, antibody, flow cytometry, vaccine, phagocytosis

## Abstract

Globally, an estimated 131 million new cases of chlamydial infection occur annually. *Chlamydia trachomatis* infection can cause permanent damage to the fallopian tubes in woman, resulting in infertility and a risk of ectopic pregnancy. There is a great need for a vaccine against *Chlamydia trachomatis* and as a result there is a need for assays to evaluate functional immune responses for use in future clinical trials and epidemiological studies. Antibodies play a crucial role in the defense against infection and can be protective by several functions, including phagocytosis and neutralization. Vaccine development could greatly benefit from a method to measure functional *C. trachomatis*‐specific antibodies in a large number of samples. In the current in vitro antibody protection assays, which measure the capacity of antibodies to facilitate phagocytic uptake of *C. trachomatis*, the phagocytosed bacteria have to be counted manually. This is both labor demanding, time consuming, and it prevents high‐throughput usage of this method. In this study, we, therefore, developed a simple and rapid flow cytometry based assay to measure the capacity of antibodies to mediate Fc‐receptor dependent phagocytosis. This method is highly reproducible and suitable to analyze large numbers of clinical and nonclinical samples. © 2018 The Authors. Cytometry Part A Published by Wiley Periodicals, Inc. on behalf of ISAC.

Chlamydiaceae are a family of gram‐negative intracellular bacteria with a wide host range and diverse pathological outcomes 1. Globally, an estimated 131 million new cases of *Chlamydia trachomatis* (*C. trachomatis)* infections occur annually. Genital infection with *C. trachomatis* (Sv D‐K and L) is the most common bacterial sexually transmitted infection. Ascension of chlamydial infection to the female upper genital tract can cause acute pelvic inflammatory disease, infertility, and ectopic pregnancy. Shortcomings of current chlamydia control strategies, has highlighted the need for a vaccine against *C. trachomatis* 2.

Protective immunity against an infection with *C. trachomatis* involves both T and B cells 3, 4, 5. As a consequence, development of high‐throughput assays for determination of T cell‐specific epitopes, as well as B cell specific epitopes is important. Recently, the role of neutralizing antibodies (Abs) has received increasing attention, and immunization with an extended major outer membrane protein (MOMP) variable domain 4 (VD4) region, containing the conserved LNPTIAG region, elicited neutralizing Abs in mice 6. However, although recent reports have demonstrated that neutralizing Abs can be protective against infection with *C. trachomatis* 6, other studies have indicated that neutralization may not be sufficient for protection 7, 8, 9, 10. Thus, it has been suggested that Abs may exert several roles in addition to neutralization during an infection with *C. trachomatis* 11. Such roles could be to increase T cell activation, induce killing of epithelial cells via antibody‐dependent cell‐mediated cytotoxicity 12, 13 or phagocytosis of antibody‐coated chlamydiae 14.

Ab induced phagocytosis is mediated by the constant part of the Ab, which binds to Fc‐receptors on the surface of the phagocytic cell. A phagocytosis assay measures the ability of an Ab to increase uptake of the bacteria into a phagocytic cell, and counting of the engulfed bacteria often involves visualization via immunofluorescence staining, followed by manually counting the bacterial inclusions via microscopy. This method of bacterial counting is a time consuming, labor demanding, low‐throughput method, and only a limited number of samples can be handled. Efficient vaccine antigen discovery and development require more effective high‐throughput assays.

Here we present a simple, rapid flow cytometry (FCM) based assay to measure the capacity of Abs to mediate Fc‐receptor dependent phagocytosis. This method is highly reproducible and suitable to analyze large numbers of clinical and nonclinical samples.

## Materials and Methods

### Cells

Hela 229 cells were obtained from American Type Culture Collection (ATCC, Manassas, Virginia). The adherent cells were maintained in RPMI 1640 (Gibco^®^, Thermofischer Scientific, Waltham, Massachusetts) supplemented with 1% (vol/vol) L‐Glutamine, 1% (vol/vol) HEPES, 1% (vol/vol) nonessential amino acids (NEAA), 1% (vol/vol) pyrovate, 10 µg/ml gentamicin and 5% heat‐inactivated fetal bovine serum (FBS) at 37°C/5% CO_2_. Cells were splitted after reaching a confluence of 80%.

McCoy cells were used to determine the concentration of the bacteria used in the experiments. McCoy cells were obtained from ATCC and maintained in RPMI 1640 supplemented with 1% (vol/vol) L‐Glutamine, 1% (vol/vol) HEPES, 1% (vol/vol) pyrovate, 1% (vol/vol) NEAA, 70 µM Mercaptoethanol, 10 µg/ml gentamicin and 10% heat‐inactivated FBS at 37°C/5% CO_2_.

PLB‐985 cells, a human myeloid leukaemia cell line, were obtained from German Collection of Microorganisms and Cell Cultures (DSMZ, Braunschweig, Germany). The cells were maintained in suspension in RPMI 1640 supplemented with 1% (vol/vol) L‐Glutamine, 1% (vol/vol) HEPES, 1% (vol/vol) pyrovate, 1% (vol/vol) NEAA, 10 µg/ml gentamicin, and 10% heat‐inactivated FBS at 37°C/5% CO_2_. The culture media was changed every 3–4 days to maintain a cell density between 2 × 10^5^ and 1 × 10^6^ cells/ml. To differentiate them into a neutrophil‐like cell type, 200,000 cells/ml were stimulated with 100 mM N,N‐Dimethylformamide (DMF; Sigma‐Aldrich, St. Louis, Missouri) in culture media without HEPES and antibiotics for 5 days at 37°C/5% CO_2_. The assay media of the PLB‐985 cells was culture media without HEPES and antibiotics.

HL‐60 cells, a human promyeloblast cell line, were obtained from ATCC. These cells were maintained and stimulated identical to the PLB‐985 cells.

### Bacteria

*C. trachomatis* SvD (UW‐3/Cx; ATCC VR‐885) were propagated in Hela 229 cells and purified as described elsewhere 15. Bacteria were resuspended in sucrose (1 M)—phoshphate (0.5 M)—glutamate (0.2 M) buffer (SPG) and stored at −80°C.

### CFSE Staining

3.37 × 10^9^ IFU of SvD bacteria were washed in PBS at 20,000 g, 4°C for 20 min. The pellet was resuspended in 500 µl of a 2 µM carboxyfluorescein diacetate succinimidyl ester (CFDA SE) solution (Vybrant^®^ CFDA SE Cell Tracer Kit, Thermofischer Scientific; diluted in PBS) and incubated for 30 min at 37°C. The CFDA SE solution was prewarmed to 37°C. CFDA SE is cell permeable and as soon as it enters cells, its acetate group cleaved by intracellular esterases to form the amin‐reactive fluorescent product carboxyfluorescein succinimidyl ester (CFSE). To quench unbound CFSE, 500 µl of ice‐cold PBS containing 10% BSA was added, followed by a centrifugation at 20,000 *g*, 4°C for 20 min. The pellet was washed once more in ice‐cold PBS containing 10% BSA. The bacteria were fixed by resuspending them in 500 µl of 4.2% formaldehyde (Cytofix^®^, BD, San Jose, CA). After 20 min incubation at 4°C the bacteria were washed with PBS. The pellet was resuspended in 250 µl PBS and stored at 4°C until usage. 50% of the originally amount of SvD bacteria is lost due to the staining procedure.

### Antibodies

Anti‐Hirep1 rabbit Ab [anti‐serum against the vaccine construct Hirep1, consisting of the VD4 region from *C. trachomatis* serovar D, E, F, fused together 6], anti‐CT043 rabbit Ab (serum), anti‐CFP10 Rabbit Ab (serum) (CFP10 is a tuberculosis antigen), Mouse anti‐VD4pep4 Ab (serum), mouse anti‐CTH522 Ab, anti‐SvD Ab (serum taken from infected B6C3F1 mice), mouse anti‐chlamydia trachomatis LPS monoclonal Ab, IgG2a (Abnova, Taipei City, Taiwan; Cat. MAB6167, clone CL21–331.1), human serum (from an exposed and a nonexposed individual) described previously 16, 17, goat anti‐mouse‐IgG Alexafluor™647 conjugated IgG (Thermofischer Scientific; Cat. A21235), mouse anti‐human CD16—FITC conjugated IgG1 (BD, San Jose, CA; Cat. 560996, clone 3G8), mouse anti‐human CD32 – PE‐Cy7 conjugated IgG1 (Thermofischer Scientific; Cat. 25–0329‐41, clone 6C4), mouse anti‐human CD64—PerCP‐Cy™5.5 conjugated IgG1 (BD, San Jose, CA; Cat. 561194, clone 10.1).

### FCM Based Phagocytosis Assay

The assay was performed in a 96 U‐well Nunclon^TM^ delta surface plate (Thermofischer Scientific) with a total volume of 200 µl. CFSE‐labeled SvD bacteria and serum samples were diluted in PLB‐985 assay media, mixed 1:1 and incubated for 40 min at 37°C on a rocker table. 100 µl of DMF‐stimulated PLB‐985 cells at a concentration of 2 × 10^6^ cells/ml were then mixed with 40 µl of the bacteria‐serum mix. Assay medium was added to each well up to a total volume of 200 µl. The 96 U‐well assay plate was incubated for 4 h at 37°C on a rocker table. Afterwards, cells were immediately washed with PBS and kept at 4°C from there on.

For some controls, the stimulated PLB‐985 cells were preincubated for 30 min with different dilutions of human Fc receptor binding inhibitor monoclonal Ab (Thermofischer Scientific, Cat. 14–9161‐73) or with 20 µl of the actin inhibitor Cytochalasin D (Sigma‐Aldrich, St. Louis, MO) before the addition of bacteria‐serum mix.

### FCM Analysis to Determine Phagocytosis

All samples were measured with a BD FACSCanto equipped with a high throughput sample reader (HTS). Acquiring Software was BD FACSDIVA version 8.0.1. Analysis of the FCS files were performed with FlowJo version 10.3 (FlowJo, LLC, Ashland, Oregon).

The entire staining procedure was performed at 4°C. The PBS was removed and the cells were resuspended in 50 µl fixable viability dye eFluor^®^ 780 (Thermofischer Scientific; Cat. 65–0865‐14). After 15 min cells were washed with FACS‐buffer (PBS with 2% FBS, 0.1% sodium azide, 1 mM EDTA). The cells were then fixed for 20 min with BD Cytofix^®^ (containing 4.2% formaldehyde) and washed in PBS. Finally, the cells were resuspended in 130 µl PBS. 80 µl of the stained samples were acquired with the HTS. PLB‐985 cells were gated on FSC‐A versus SSC‐A. Doublets and triplets were excluded by gating on FSC‐A versus FSC‐H. Dead cells were excluded by gating on the negative population on the APC‐Cy7 channel. The CFSE signal was then measured in the FITC channel. The baseline for chlamydia‐positive cells was set by controls that incubated the phagocytic cells only with the CFSE‐labeled bacteria and without any serum.

### Ethical Statement

The protocol and procedures employed were reviewed and approved by our institutional review committee. Regarding the two human Abs used in the study, they have been described previously 17 in a study that was approved by the Local Ethical Committee for Copenhagen (01– 008/03). The procedures followed were in accordance with the ethical standards of Local Ethical Committee for Copenhagen on human experimentation and with the Helsinki Declaration of 1975, as revised in 2008.

## Results

### Labeling *C. trachomatis* with CFSE

To make the phagocytosis assay as simple as possible, we decided to use fluorescently labeled bacteria, thus avoiding the step of intracellular staining of phagocytosed bacteria.

**Figure 1:**
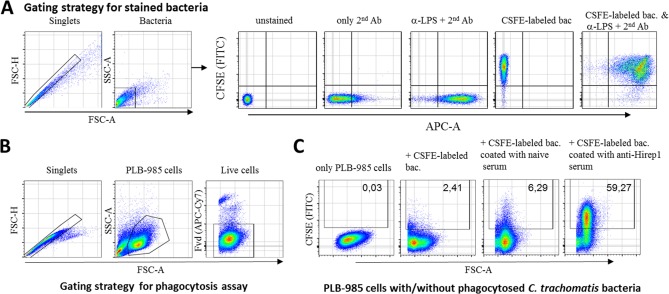
Gating strategies for CFSE‐labeled *C. trachomatis* bacteria and phagocytosis assay. All gatings were first set on singlet events (FSC‐A vs FSC‐H), followed by gating on target cell population (FSC‐A vs. SSC‐A). **(A)** Bacteria were then shown in APC‐A versus FITC‐A (stained either with mouse anti‐*C. trachomatis* LPS monoclonal Ab and a goat anti‐mouse‐IgG‐Alexaflour™647 secondary Ab or labeled with CFSE). **(B)** PLB‐985 cells were stained with fixable viability dye eFluor^®^ 780 (FvD) and gated on living cells with FSC‐A versus APC‐Cy7‐A. **(C)** CFSE‐labeled SvD bacteria were preincubated with no serum, serum from naïve rabbits, or serum from rabbits vaccinated with Hirep1 for 40 min at 37°C and incubated for 4 h with DMF‐stimulated PLB‐985 cells. CFSE‐signal was measured by flow cytometry in the FITC channel. Cells were gated on CFSE‐positive (=phagocytosing) events. Pseudo‐color dot plots show PLB‐985 cells alone or after incubation with noncoated or coated bacteria.

The first objective was to label the bacteria, and measure the labeling by flow cytometry. To label the bacteria, we used CFSE (as explained in materials and methods). Labeling with CFSE occurs inside the bacteria, making it a good method to visualize antibody mediated phagocytosis of bacteria, as it does not interact with surface antigens. At first, *C. trachomatis* (SvD) bacteria were stained with CFSE and analyzed by flow cytometry. The gating strategy is shown in Figure 1A. For comparison, bacteria were stained with an anti‐*C. trachomatis* LPS Ab and some were co‐stained with the anti‐*C. trachomatis* LPS Ab and CFSE‐labeled. The data showed an efficient labeling with CFSE, and an almost complete co‐staining of anti‐*C. trachomatis* LPS on CFSE‐labeled bacteria (Fig. 1A). Moreover, CFSE labeling and fixation did not affect phagocytosis of the bacteria, as we observed similar phagocytosis of CFSE labeled and nonlabeled bacteria (Supporting Information Fig. S1).

### Antibody Mediated Uptake of Bacteria

We next coated the CFSE labeled SvD bacteria with different concentrations of rabbit polyclonal Abs directed against Hirep1. The Hirep1 vaccine construct is based on the *C. trachomatis* Major Outer Membrane Protein (MOMP), and is known to induce neutralizing antibodies [6 and Olsen et al. unpublished observations)]. We measured the uptake of Ab‐coated bacteria by PLB‐985 cells, an immature myeloid cell line, which was differentiated by DMF treatment for 5 days into terminally mature neutrophils, closely mimicking the functions of blood neutrophils 18. The measurement was performed by flow cytometry and the gating strategy for measuring uptake of CFSE labeled bacteria into PLB‐985 cells is shown in Figure 1B.

**Figure 2:**
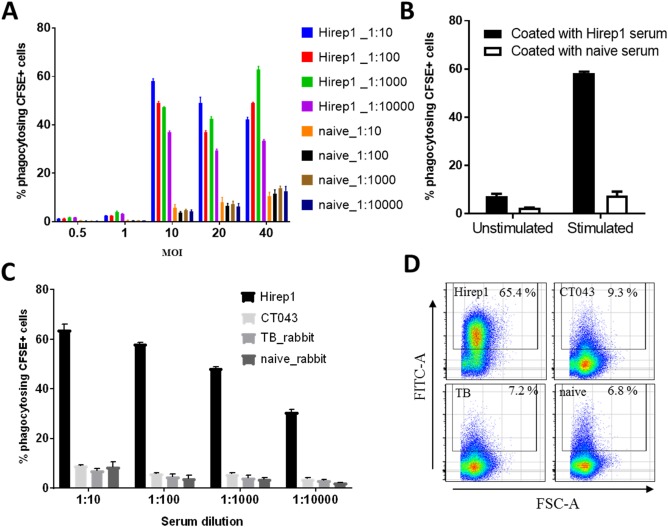
Antibody mediated phagocytosis. CFSE‐labeled SvD bacteria were pre‐incubated with serum of vaccinated and naïve rabbits for 40 min at 37 °C and incubated for 4 h with DMF‐stimulated PLB‐985 cells. CFSE‐signal was measured by flow cytometry in the FITC channel. Cells were gated on CFSE‐positive (=phagocytosing) events. **(A)** Bacteria were titrated from MOI 0.5 to MOI 40 and sera from naïve and Hirep1‐vaccinated rabbits were titrated from a 1:10 to a 1:10000 dilution. **(B)** Unstimulated and DMF‐stimulated PLB‐985 cells were incubated with serum‐coated CFSE‐labeled SvD bacteria at MOI 10. **(C)** SvD bacteria at MOI 10 were incubated with different rabbit sera that were titrated 1:10 to 1:10000. Phagocytosis was measured by flow cytometry. **(D)** Representative pseudo‐color dot plots of DMF‐stimulated PLB‐985 cells in FSC‐A vs FITC‐A with different rabbit sera at a dilution of 1:10 and SvD bacteria at MOI of 10. Mean and SD at a sample size of *n* = 3 are shown (A to C).

Control stainings are shown in Figure 1C. It shows a comparison of PLB‐985 cells with PLB‐985 cells being subjected to CFSE‐labeled *C. trachomatis* bacteria (noncoated or precoated with anti‐Hirep1 serum or naïve serum). Although some phagocytosis was observed by adding CFSE labeled bacteria (noncoated or coated with naïve serum) to PLB‐985 cells, the phagocytosis increased when adding *C. trachomatis* bacteria that had been coated with anti‐Hirep1 serum (Fig. 1C). At MOIs of 10, 20, or 40, anti‐Hirep1 Abs induced a strong phagocytosis. Approximately 58% of PLB‐985 cells stained positive for *C. trachomatis* (at MOI 10) (Fig. 2A). Decreasing the concentration of the Abs led, as expected, to a decrease in phagocytosis to 37% at a serum dilution of 1:10,000. Significantly less positive cells were observed in the presence of serum from naïve animals (Fig. 2A). We choose a MOI of 10 as the bacteria‐to‐cell ratio we would use in this assay. Furthermore, we noted that the phagocytosing capability of the PLB‐985 cells increased following stimulation with DMF (Fig. 2B). To determine the variability of the phagocytosis assay it was repeated in five independent experiments with anti‐Hirep1 Ab coated CFSE‐labeled bacteria at a serum dilution of 1:10 and bacteria concentration of MOI 10 with DMF stimulated PLB‐985 cells. The intra‐assay coefficient of variation (CV) was between 1.13 and 2.77% and the interassay CV was 3.76% (Table 1). We, therefore, conclude that the assay is reproducible using a serum dilution of 1:10 and bacteria concentration of MOI 10. Using serum dilutions of 1:100–1:10,000 gave, as expected, a reduced phagocytosis and a higher CV value, although even with a dilution of 1:10,000, the assay was reproducible (Supporting Information Fig. S2).

**Table 1:** Intra and Interassay coefficient of variation

	% Phagocytosing CFSE+ PLB‐985 cells					
Exp. number	Replicate 1	Replicate 2	Replicate 3	Mean	SD	Intra CV (%)
1	57.4	57.4	59.2	58.00	0.85	1.46
2	65.4	65.1	61.5	64.00	1.77	2.77
3	62.3	62.9	61.2	62.13	0.70	1.13
4	60.4	62.2	59.5	60.70	1.12	1.85
5	59.3	57.1	58.5	58.30	0.91	1.56
			Inter CV (%)	3.76		

We next compared the anti‐Hirep1 Ab with a rabbit antiserum directed against another chlamydial surface‐exposed antigen, the CT043 antigen 19. Interestingly, in contrast to the anti‐Hirep1 Ab, an Ab directed against the CT043 antigen did not induce phagocytosis, demonstrating that being surface exposed does not automatically lead to phagocytosis. We also tested a control serum from rabbits vaccinated with a tuberculosis antigen [“H56” 20]. As expected, this Ab did also not induce phagocytosis (Figs. 2C and 2D).

**Figure 3:**
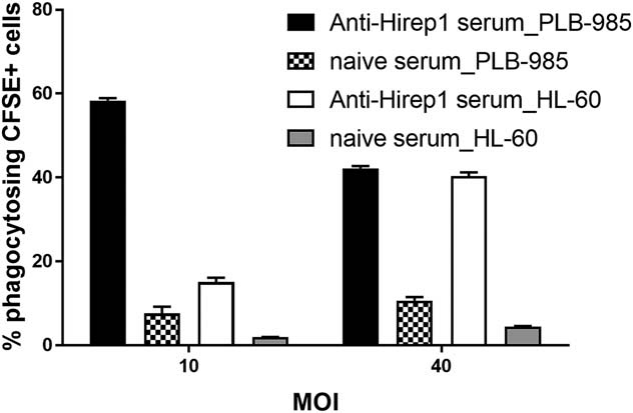
Comparison of phagocytosis by PLB‐985 and HL‐60 cells. CFSE‐labeled SvD bacteria at MOIs of 10 and 40 were incubated with 1:10 diluted sera of naïve and Hirep1 vaccinated rabbits. DMF‐stimulated PLB‐985 and HL‐60 cells were then incubated with the coated bacteria. Phagocytosis was measured by flow cytometry. Mean and SD are shown at a sample size of *n* = 3.

Finally, we also tested the FCM assay with HL‐60 cells, another neutrophilic cell line that is used in several laboratories. CFSE labeled bacteria coated with the anti‐Hirep1 Ab were added to DMF stimulated HL‐60 cells and phagocytosis was measured by FCM, as explained above. The results showed that at a MOI of 40 HL‐60 and PLB‐985 cells showed a similar phagocytosis (Fig. 3). However, at a MOI of 10, PLB‐985 cells showed a significantly higher phagocytosis compared to HL‐60 cells.

Taken together, using a rabbit Ab specific for MOMP, we could show phagocytosis of the CFSE labeled bacteria. The percentage of phagocytosing cells was dependent on the amount of Ab used. Compared to the naïve rabbit control serum, no increase in phagocytosis was observed with antibodies directed against a tuberculosis antigen or with an anti‐CT043 Ab.

### Inhibition of Phagocytosis

**Figure 4:**
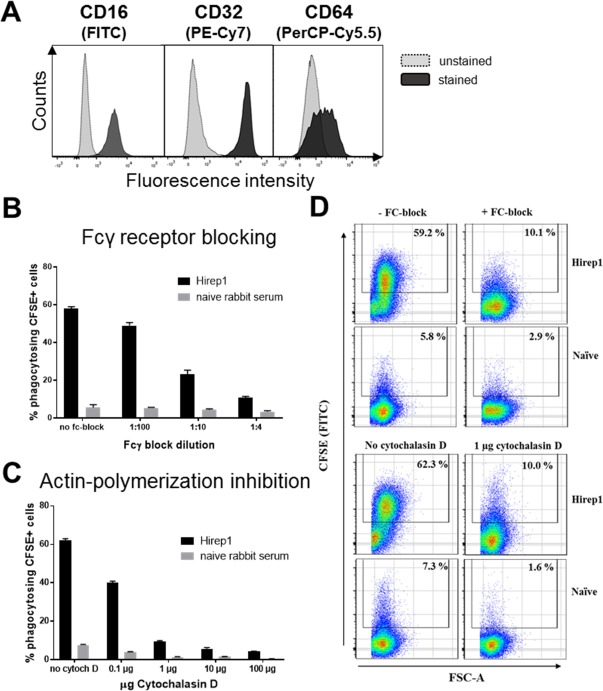
Dependence of phagocytosis on Fcγ receptors and actin polymerization. **(A)** Expression of CD16, CD32 and CD64 on the surface of DMF stimulated PLB‐985 cells. **(B)** CFSE‐labeled SvD bacteria at a MOI of 10 were incubated with 1:10 diluted sera of naïve and Hirep1 vaccinated rabbits and given to DMF stimulated PLB‐985 cells that were either pre‐incubated with different dilutions of human Fcγ‐receptor‐block (1:4, 1:10, 1:100), or with **(C)** different concentrations of Cytochalasin D (0.1 μg, 1 μg, 10 μg, 100 μg); **(D)** Phagocytosis of PLB‐985 cells was measured by flow cytometry. Representative pseudo‐color dot plots of treatment with Fcγ‐receptor‐block (1:4) or Cytochalasin D (1 μg) in comparison to untreated PLB‐985 cells. Mean and SD are shown at a sample size of *n* = 3 (B and C).

We next wanted to explore the mechanism behind the observed phagocytosis. As expected, the Fcγ receptors (CD16 and CD32) were expressed on PLB‐985 cells and CD64 on ∼40% of the cells (Fig. 4A). To confirm that the phagocytosis was dependent on Fcγ receptors, we examined the effect of inhibiting the binding of anti‐Hirep1 Abs to the Fcγ receptors, on the ability of anti‐Hirep1 Abs to induce phagocytosis. Stimulated PLB‐985 cells were preincubated with three different dilutions of human Fcγ receptor binding inhibitor polyclonal Ab (1:4, 1:10, 1:100), prior to incubation with CFSE‐labeled bacteria coated with the anti‐Hirep1 Ab. The result showed that in the absence of preincubation with Fcγ receptor inhibitor, approximately 60% of the PLB‐985 cells were SvD positive (Fig. 4B). Preincubation with Fcγ receptor inhibitor reduced the phagocytosis in a concentration dependent manner (Figs. 4B and 4D) from 60% to 11%. Thus, efficient phagocytosis was dependent on an intact interaction between the Ab coated bacteria and the Fcγ receptors.

Several reports have shown that Fc receptor mediated internalization involve polymerization of actin 21, 22. Therefore, preventing actin polymerization should inhibit internalization. To test this, PLB‐985 cells were preincubated with Cytochalasin D, an inhibitor of actin polymerization, before the addition of bacteria‐serum mix. At all concentrations tested, Cytochalasin D blocked Ab mediated phagocytosis of the labeled bacteria (Figs. 4C and 4D).

In summary, PLB‐985 cells stained positive for CD16 and CD32 and partially for CD64. Moreover, using the FCM phagocytosis assay, we show that anti‐Hirep1 Ab induced phagocytosis could be blocked by an Fcγ receptor inhibitor, or by inhibiting the polymerization of actin.

### Phagocytosis Mediated by Human and Murine Serum

An obvious use for a FCM based phagocytosis assay is the high‐throughput testing of serum samples from human donors, or samples from mice vaccinated with different vaccine candidates. It was, therefore, important to show that the assay is also suitable for human and murine serum. We used serum from mice infected with *C. trachomatis* SvD and serum from mice immunized with a construct containing the neutralizing epitope in the VD4 region of MOMP, “VD4p4.” In addition, we tested a murine monoclonal Ab specific for *C. trachomatis* LPS.

The assay was performed in the same way as shown above with the rabbit serum. CFSE‐labeled SvD *C. trachomatis* bacteria were coated with diluted serum samples (1:10–1:10,000), and subsequently incubated with the DMF‐stimulated PLB‐985 cells for 4 h, before being subjected to FCM analysis.

**Figure 5:**
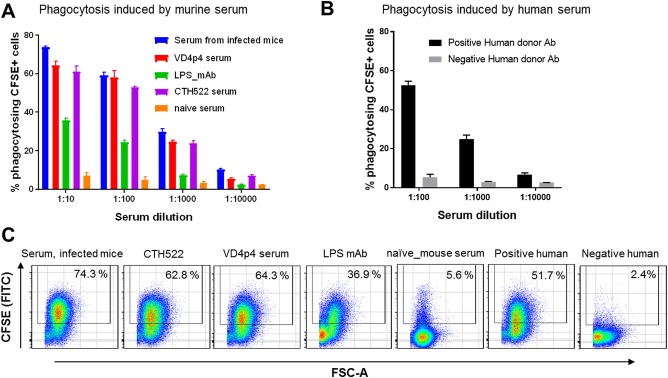
Phagocytosis assay using human and murine serum. CFSE‐labeled SvD bacteria at a MOI of 10 were incubated with **(A)** serum from infected/vaccinated mice or a monoclonal mouse‐a‐*C. trachomatis* LPS Ab, or **(B)** serum from an infected human donor. Serum of naïve mice/humans were used as negative controls. Murine sera were titrated from 1:10 to 1:10000 (*C. trachomatis* LPS mAb was pre‐diluted to 1 ng/μl) and human sera from 1:100 to 1:10000. Phagocytosis was measured by flow cytometry. **(C)** Representative pseudo‐color dot plots of the phagocytosis assay with all sera at a dilution of 1:10 (for human sera 1:100). Mean and SD are shown at a sample size of *n* = 3 (A and B).

Both serum from infected and immunized mice induced phagocytosis (Fig. 5A). A murine anti‐*C. trachomatis* LPS mAb also induced phagocytosis in a dose‐dependent manner (Fig. 5A).

We also tested serum from a human donor with a confirmed genital SvD infection. We choose a donor that we have previously shown to possess Abs directed against MOMP SvD 16, 17. In contrast to serum from a nonexposed “naïve” human donor, the serum from the exposed human donor induced a strong phagocytosis leading to 52.6% positive PLB‐985 cells (Fig. 5B).

Taken together, the assay showed to be applicable for both human and murine serum. Murine Abs directed against the VD4 region from MOMP induced phagocytosis of the *C. trachomatis* bacteria, whereas serum from nonvaccinated mice did not. Anti‐LPS Abs also induced phagocytosis.

## Discussion

The flow cytometry assay was recently shown to be an alternative to microscopy in terms of counting and titrating bacteria 23. In this article, we show that flow cytometry is also suitable for high‐throughput testing of the phagocytosing ability of an Ab directed against an intracellular bacterium, in our case *C. trachomatis*.

For the FACS based phagocytosis assay, we used CFSE labeled bacteria. While others have also used CFSE labeling to visualize the appearance of bacteria in target cells 24, there are other options, such as fluorescein isothiocyanate (FITC) or a pH sensitive dye pHrodo™ 25, 26. FITC is an amine group reactive compound that binds to every protein, which is also true for CFSE. However, we used CFDA SE, which is a cell permeable dye. Once inside the bacteria, intracellular esterases cleave the acetate groups, which results in the reactive CFSE form. Thus, this method provides an intrabacterial labeling, while FITC stains the outside of the bacteria. The pH sensitive pHrodo dye is labeling the bacteria in the same way as CFDA SE. However, the dye will change its excitation maximum according to the pH, and this allows a distinction between bacteria in a neutral pH environment, such as on the surface of cells, or bacteria in an acidic intracellular environment, such as the phagolysosomes. In future experiments, we will compare the different staining methods. It should be noted that the pHrodo dye in general is more expensive than CFDA SE, which is an important factor to consider in the development of a high throughput assay, to be used with many samples.

We observed 50–70% of phagocytosing cells after coating bacteria with serum of vaccinated or infected animals/individuals in our in vitro assay. Phagocytosis was observed not only with human serum, but also with murine and rabbit serum. The ability of IgG from different species to bind to human Fc receptors is in agreement with previous studies 27, 28, 29, 30. Fabbrini et al. developed an Ab dependent phagocytosis assay for Group B Streptococcus 26. They used the human neutrophil‐like HL‐60 cell line. They also tested different murine and rabbit sera from vaccinated animals and achieved a phagocytic uptake of approximately 60%. We also tested the HL‐60 cell line, and found HL‐60 cells also efficiently phagocytosed Ab coated bacteria, although requiring higher MOIs than with PLB‐985 cells. The specificity and sensitivity of the FCM‐based assay was not compared with the standard microscopy assay.

The phagocytosis in our assay was Fcγ receptor and actin polymerization dependent as blocking FcR binding or actin polymerization prevented uptake of the bacteria (Fig. 4). The FcR blocking reagent is known to bind Fcg receptors, indicating a role for these receptors in mediating the phagocytosis. All the Fcγ receptors are capable of endocytosis, and the precise role for each of the individual Fcγ receptors in phagocytosing *C. trachomatis* requires further experiments 31, 32. Interestingly, the FACS assay could distinguish between a binding nonphagocytosing Ab (directed against CT043) and a binding phagocytosing Ab (directed against Hirep1) (Fig. 2C). As phagocytosis require crosslinking of the Fc receptor 33, it could be speculated that the lack of phagocytosis with the CT043 Ab is due to the CT043 antigen being too far apart on the chlamydial surface for the binding Abs to crosslink the Fc receptor on the phagocyte.

We also tested an LPS mAb and found that despite a complete lack of neutralizing capability (data not shown), this mAb was, however, able to induce phagocytosis of *C. trachomatis* (Fig. 5). Thus, this mAb could be used to selectively study the phagocytosis of *C. trachomatis*.

In conclusion, we have developed a simple, rapid, reproducible flow cytometric based assay that use cultivable effector/target cells and is able to handle a large number of samples.

## Supporting information

Additional Supporting Information may be found in the online version of this article

Supporting MIFlowCyt

Supporting Figure1

Supporting Figure2

Supporting Figure Legends
