# Supplementary material for: A flow cytometry‐based assay to determine the phagocytic activity of both clinical and nonclinical antibody samples against Chlamydia trachomatis
Source: Cytometry A. 2018 Mar 7;93(5):525–32. doi: 10.1002/cyto.a.23353 (PMC6033180; doi:10.1002/cyto.a.23353)
Supplement: Supplementary file 1 — Supporting MIFlowCyt [file CYTO-93-525-s001.doc]

**Cytometry Part A**

**Author Checklist: MIFlowCyt-Compliant Items**

| **Requirement** | **Please Include Requested Information** |
| --- | --- |
| 1.1. Purpose | The purpose of this work was to develop a high-throughput flow-cytometry based assay to determine phagocytic capacity of clinical and pre-clinical serum samples against *Chlamydia trachomatis*. |
| 1.2. Keywords | Chlamydia, antibody, Flow cytometry, vaccine, phagocytosis |
| 1.3. Experiment variables | - Concentration of CFSE-labeled *C. trachomatis* (MOI 0.5, 1, 10, 20, 40) - Serum dilutions (final dilutions of 1:10, 1:100, 1:1000, 1:10000) - Cell-line (PLB-985, HL-60) |
| 1.4. Organization name and address | Statens Serum Institut, Department of Infectious Disease Immunology  Address: Artillerivej 5, DK-2300 Copenhagen S, Denmark |
| 1.5. Primary contact name and email address | Jes Dietrich  jdi@ssi.dk |
| 1.6. Date or time period of experiment | June to August 2017 |
| 1.7. Conclusions | - MOI 10 is the chosen bacteria concentration to phagocytosis - PLB-985 cells show increased phagocytosing capability compared to HL-60 cells - Uptake of bacteria in our assay is Fcγ receptor and actin polymerization dependent => phagocytosis - Assay is highly reproducible (intra- & inter-assay CV below 5%) - Our FACS phagocytosis assay can distinguish between a binding non-phagocytosing antibody and a binding phagocytosing Ab - The assay can be used with serum from rabbits, mice and humans - the assay is able to handle large numbers of samples (High through-put) |
| 1.8. Quality control measures | - The assay was always performed with serum from naïve animals / non-infected humans - PLB-985 cells were incubated with human Fcγ receptor-inhibitor prior to incubation with serum-coated bacteria to confirm role of FcR (see also Fig 4B & D) - PLB-985 cells were incubated with cytochalasin D prior to incubation with serum-coated bacteria to confirm role of actin polymerization (see also Fig 4C & D) |
| 2.1.1.1. (2.1.2.1., 2.1.3.1.) Sample description | - Cells: PLB-985 (from DSMZ) & HL-60 cells (from ATCC) were stimulated with 100 mM DMF for 5 days and 200.000 cells were plated in 96-U-well plates (concentration of 2 mio/ml) - Bacteria: *C. trachomatis* SvD bacteria were labeled with CFSE (see Material & Methods) and stored for up to 4 weeks in PBS at 4 °C - Serum: samples from vaccinated/infected/naïve/non-infected animals/humans were heat-inactivated for 30 min at 56 °C |
| 2.1.1.2. Biological sample source description | Bacteria: *C. trachomatis* SvD (UW-3/Cx; ATCC VR-885)  Human serum: Two human Abs (from a *Chlamydia trachomatis* exposed and a non-exposed individual) described previously (ref. 15,16 in paper – see also 2.1.1.3)  Mouse serum: Mouse VD4pep4 Ab produced at SSI in B6C3F1 mice , mouse CTH522 Ab produced at SSI in B6C3F1 mice, anti-SvD Ab from infected B6C3F1 mice  Rabbit serum (New Zealand White Rabbits): Hirep1 rabbit Ab (VD4 region from serovar D, E, F, described in Olsen AW, Follmann F, Erneholm K, Rosenkrands I, Andersen P. Protection against Chlamydia trachomatis infection and upper genital tract pathological changes by vaccine-promoted neutralizing antibodies directed to the VD4 of the major outer membrane protein. Journal of Infectious Diseases 2015:jiv137),  CT043 rabbit Ab produced at SSI in New Zealand White Rabbits, tuberculosis antigen CFP10 Rabbit Ab produced at SSI in New Zealand White Rabbits |
| 2.1.1.3. Biological sample source organism description | Human Abs have been described here:  Follmann F, Olsen AW, Jensen KT, Hansen PR, Andersen P, Theisen M. Antigenic profiling of a Chlamydia trachomatis gene-expression library. Journal of Infectious Diseases 2008;197:897-905, and  Olsen AW, Follmann F, Højrup P, Leah R, Sand C, Andersen P, Theisen M. Identification of human T-cell targets recognized during the Chlamydia trachomatis genital infection. J Infect Dis 2007;196:1546-1552.  Mouse: B6C3F1 mice  Rabbit : New Zealand White Rabbits |
| 2.1.2.2. Environmental sample location | NA |
| 2.3. Sample treatment description | The assay was performed in a 96 U-well NunclonTM delta surface plate (Thermofischer Scientific, St. Louis, USA) with a total volume of 200 µl. CFSE-labeled SvD bacteria and serum samples were diluted in PLB-985 assay media, mixed 1:1 and incubated for 40 min at 37 °C on a rocker table. 100 µl of DMF-stimulated PLB-985 cells at a concentration of 2 mio/ml were then mixed with 40 µl of the bacteria-serum mix. Assay media was then added to a total volume of 200 µl. The 96 U-well assay plate was incubated for 4 h at 37 °C on a rocker table. Afterwards, cells were washed with PBS and kept at 4 °C from there on. The PBS was removed and the cells were resuspended in 50 µl fixable viability dye eFluor® 780 (Thermofischer Scientific, St. Louis, USA; Cat. 65-0865-14). After 15 min cells were washed with FACS-buffer (PBS with 2 % FBS, 0.1 % sodium azide, 1 mM EDTA). The cells were then fixed for 20 min with BD Cytofix® (containing 4.2 % formaldehyde) and washed in PBS. Finally, the cells were resuspended in 130 µl PBS. 80 µl of the stained samples were acquired with the high-throughput sample reader (HTS).  For additional information see materials and methods |
| 2.4. Fluorescence reagent(s) description | Each sample has been stained with a fixable viability dye eFluor® 780 (Thermofischer Scientific, St. Louis, USA; Cat. 65-0865-14), which appeared in the APC-Cy7 channel. The bacteria were labeled with CFSE (Vybrant® CFDA SE Cell Tracer Kit, Thermofischer Scientific, St. Louis, USA; diluted in PBS), which appeared in the FITC channel.  Compensation was unnecessary and thus not performed.  Fcγ receptor expression on PLB-985 cells was examined with the following Abs: mouse anti-human CD16 – FITC conjugated Ab (BD, San Jose, CA USA; Cat. 560996), mouse anti-human CD32 – PE-Cy7 conjugated Ab (Thermofischer Scientific, St. Louis, USA; Cat. 25-0329-41), mouse anti-human CD64 – PerCP-Cy™5.5 conjugated Ab (BD, San Jose, CA USA; Cat. 561194). Single staining was performed with those Abs, and thus compensation was also not performed.  CFSE-labeled SvD bacteria were co-stained with mouse anti-chlamydia trachomatis LPS monoclonal Ab (Abnova, Taipei City, Taiwan; Cat. MAB6167), human Ab (from infected individuals), goat anti-mouse-IgG- Alexafluor™647 conjugated Ab (Thermofischer Scientific, St. Louis, USA; Cat. A21235). Also here, compensation was not performed. |
| 3.1. Instrument manufacturer | BD Biosciences |
| 3.2. Instrument model | BD FACSCanto equipped with a high throughput sample reader (HTS)  Cat. No.: 337175, serial no: V0101 |
| 3.3. Instrument configuration and settings | - Flow cell: instrument has not been altered; rectangular quartz flowcell - Sample input: samples were acquired using a BD high throughput sampler from a 96-U-wellplate - Light sources: instrument has not been altered; two lasers air-cooled:   - - 20 mW solid state 488 nm     - 17 mW HeNe 633 nm - Excitation optics configuration: instrument has not been altered; Spatially separated beams with 9 x 65 µm elliptical spots - Optical filters: instrument has not been altered. - Optical detectors: instrument has not been altered. The Fluorescence detectors are arranged in 6 PMTs in 4+2 configuration with default blue laser dyes: FITC, PE, PerCP or PerCP-Cy5.5, PE-Cy7 (525, 575, 678 or 695, 785 nm); and default red laser dyes: APC, APC-Cy7 (660, 785 nm). The Detector voltages for the phagocytosis assay have been set to: FSC=5V; SSC=350V;FITC=500V;APC-Cy7=500V;APC=500V   For Fig 1A where bacteria were acquired alone the following detector voltages have been altered: FSC=300V; SSC=400V; FITC=450V; APC-Cy7 was not used  For Fig 4A where Fcγ receptor expression on PLB-985 cells was examined, single stainings were performed with the respective Ab & and only the detector for the Ab-coupled flourochrome was used together with FSC=5V and SSC=350V:  for CD16 => FITC=618V  for CD32 => PeCy7=621V  for CD64 => PerCP-Cy5.5=668V     - Optical paths: instrument has not been altered |
| 4.1. List-mode data files | We did not upload the FCS files. We have integrated original dot-plots into the manuscript as examples for our gating strategies and results, with the different sera we used in the phagocytosis assay. Furthermore we used only two flourochromes (FITC, APC-Cy7) in our assay.. If FCS files are required, we are happy to provide on request to Jes Dietrich |
| 4.2. Compensation description | Compensation was not performed |
| 4.3. Data transformation details | FlowJo version 10.3 (FlowJo, LLC, Ashland, USA) was used for data transformation in order to allow visualization and gating. All files have been automatically biexponetially transformed using the logicle implementation. All fluorescence parameters were displayed on a logarhythmic “logical” scaling with 0 extra negative decades and a width basis of -10.00. FSC-A, FSC-H, SSC-A, SSC-H were displayed on a linear scale. |
| 4.4.1. Gate description | All data files for the phagocytosis assay underwent identical gating strategy with identical gates   - All gatings were first set on singlet events (FSC-A vs FSC-H) - followed by gaiting on PLB-985 cell population (FSC-A vs SSC-A) - gated on living cells (FSC-A vs APC-Cy7-A) - Phagocytosing cells were then gated on FSC-A vs FITC-A based on the CFSE-signal in the target cells (the FITC-A positive cells are referred to as phagocytosing cells) |
| 4.4.2. Gate statistics | The following table shows percentages of each subpopulation defined by the described gates for the phagocytosis assay with **Hirep1 coated**, CFSE-labeled *C. trachomatis* bacteria.   | Example Hirep-1 serum | Singlets | PLB-985 | Living cells | CFSE+ cells | | --- | --- | --- | --- | --- | | Tot% | 98.7 |  |  |  | | Singlets% |  | 61.3 |  |  | | PLB-985% |  |  | 97.0 |  | | Living cells% |  |  |  | 60.9 |   The following table shows percentages of each subpopulation defined by the described gates for the phagocytosis assay with CFSE-labeled *C. trachomatis* bacteria, that have been **coated with serum from naïve** rabbits.   | Example naïve serum | Singlets | PLB-985 | Living cells | CFSE+ cells | | --- | --- | --- | --- | --- | | Tot% | 99.1 |  |  |  | | Singlets% |  | 61.2 |  |  | | PLB-985% |  |  | 96.4 |  | | Living cells% |  |  |  | 6.6 | |
| 4.4.3. Gate boundaries | 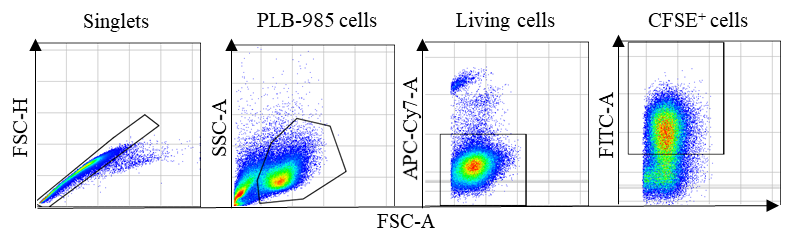  Bacteria were coated with **Hirep1 antibody.** |

**Notes**

Feel free to use more space than allocated.

You can embed graphics/figures in this document, if needed.

Please make sure to save the document in Microsoft Word version 2003 or older, before uploading to ScholarOne Manuscripts. When uploading this checklist to ScholarOne Manuscripts, please choose the “Supplementary Material for Review” category.

Please note that if your paper is accepted, the checklist will be published as an Online Supporting Information.

For any questions, please contact the Cytometry Part A editorial office at [Cytometrya@wiley.com](mailto:Cytometrya@wiley.com).
