# Supplementary figures and images for: A flow cytometry‐based assay to determine the phagocytic activity of both clinical and nonclinical antibody samples against Chlamydia trachomatis
Source: Cytometry A. 2018 Mar 7;93(5):525–32. doi: 10.1002/cyto.a.23353 (PMC6033180; doi:10.1002/cyto.a.23353)

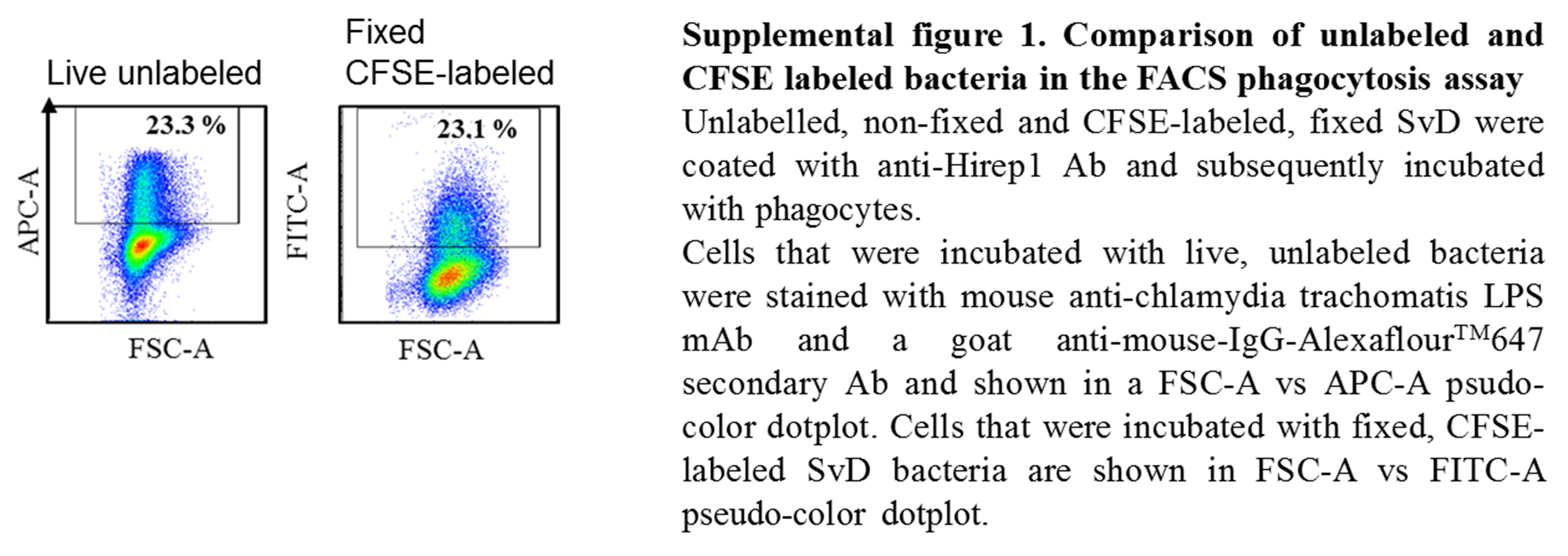

Supplement: Supplementary file 2 — Supporting Figure1 [file CYTO-93-525-s002.tif]

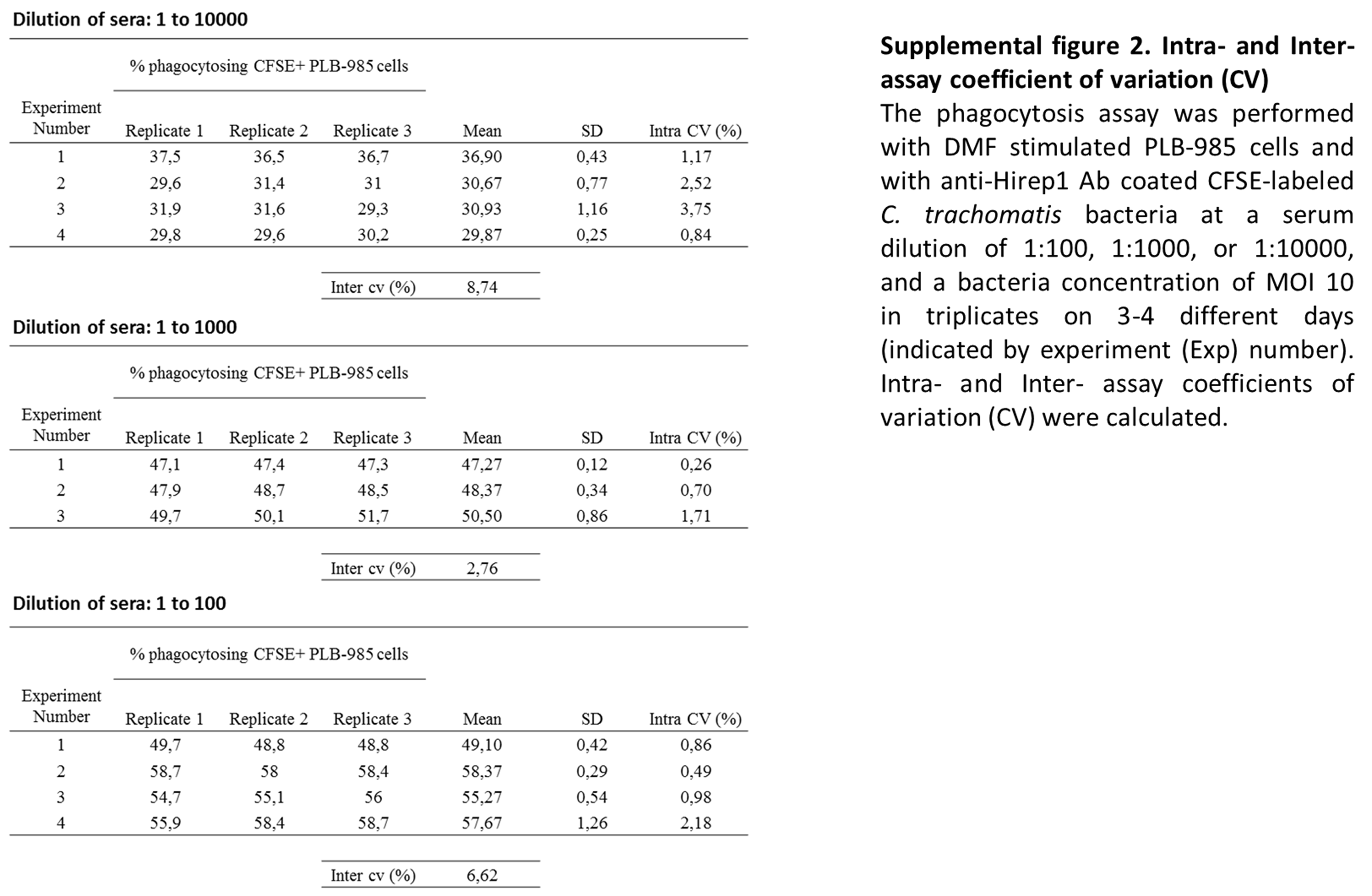

Supplement: Supplementary file 3 — Supporting Figure2 [file CYTO-93-525-s003.tif]
