# Supplementary material for: A flow cytometry‐based assay to determine the phagocytic activity of both clinical and nonclinical antibody samples against Chlamydia trachomatis
Source: Cytometry A. 2018 Mar 7;93(5):525–32. doi: 10.1002/cyto.a.23353 (PMC6033180; doi:10.1002/cyto.a.23353)
Supplement: Supplementary file 4 — Supporting Figure Legends [file CYTO-93-525-s004.docx]

**Supplemental figure 1.** Comparison of unlabeled and CFSE labeled bacteria in the FCM phagocytosis assay.

Unlabeled, non-fixed and CFSE-labeled, fixed SvD were coated with anti-Hirep1 Ab and subsequently incubated with phagocytes. Cells that were incubated with live, unlabeled bacteria were stained with mouse anti-chlamydia trachomatis LPS mAb and a goat anti-mouse-IgG-Alexaflour^TM^647 secondary Ab and shown in a FSC-A vs APC-A psudo-color dotplot. Cells that were incubated with fixed, CFSE-labeled SvD bacteria are shown in FSC-A vs FITC-A pseudo-color dotplot.

**Supplemental figure 2**. Intra- and Inter- assay coefficient of variation (CV).

The phagocytosis assay was performed with DMF stimulated PLB-985 cells and with anti-Hirep1 Ab coated CFSE-labeled C. trachomatis bacteria at a serum dilution of 1:100, 1:1000, or 1:10000, and a bacteria concentration of MOI 10 in triplicates on 3-4 different days (indicated by experiment (Exp) number). Intra- and Inter- assay coefficients of variation (CV) were calculated.
